# Cytomegalovirus Enteritis: A Rare Culprit of Protein Losing Enteropathy in an Immunocompetent Adult

**DOI:** 10.14309/crj.0000000000001768

**Published:** 2025-07-21

**Authors:** Nancy Mayer, Vijay Kata, Dhaval Patel, Daniel Errampalli, Thomas O'Connor, Matthew Kaplan, Thomas Betlej, Nikhil Bhargava

**Affiliations:** 1 Department of Internal Medicine, Riverside Medical Center, Kankakee, IL; 2 Digestive Diseases Consultants of Kankakee, Bourbonnais, IL; 3 Department of Clinical and Anatomical Pathology, Riverside Medical Center, Kankakee, IL

**Keywords:** protein-losing enteropathy, cytomegalovius, CMV enteritis, duodenitis, immunocompetent host, alpha-1 antitrypsin clearance

## Abstract

Protein-losing enteropathy (PLE) is a rare complication of cytomegalovirus (CMV) enteritis in immunocompetent individuals. We present a case of a previously healthy 60-year-old woman with an acute CMV infection, manifesting as intractable vomiting and diarrhea, refractory electrolyte disturbances, and anasarca. The diagnostic evaluation showed low serum protein levels and an increased fecal alpha-1 antitrypsin clearance, confirming PLE. CMV-specific immunohistochemistry on duodenal samples subsequently confirmed that the PLE was caused by CMV enteritis. She completed treatment with valganciclovir for 3 weeks, resulting in symptom resolution and normalization of endoscopic findings. This case highlights the importance of recognizing CMV-associated PLE, particularly in immunocompetent individuals, for timely diagnosis and treatment.

## INTRODUCTION

Protein-losing enteropathy (PLE) is a rare condition characterized by an excessive leakage of plasma proteins into the gastrointestinal tract. Excessive protein loss presents clinically with third-space fluid accumulation (ascites, pleural, and pericardial effusions) along with malnutrition. Though rare, there are diverse causes of PLE that can be divided into inflammatory disorders, disorders of mucosal permeability, and disorders involving lymphatic obstruction. Some culprit diseases include congestive heart failure, lymphoma, and inflammatory bowel disease.^1^ Cytomegalovirus (CMV) is a rarely described culprit of PLE in an otherwise healthy adult, with only 13 documented cases in literature^2–15^ (Table 1). In this study, we present a case of PLE due to acute CMV enteritis in an immunocompetent patient.

**Table 1.** Cases of CMV-associated PLE in immunocompetent adults

Reference	Age	Sex	Coexisting conditions	Presentation	Laboratory markers of systemic CMV involvement	Imaging results	Endoscopic observations	Histopathologic features	CMV diagnostic methods	Treatment	Outcome
Ciavaldini^2^	28	M	None	Abdominal pain, edema	Elevated LFTs	Gastric and small bowel inflammation, lymphadenopathy	Ulcerative and congestive gastritis	Ulcerative and edematous gastritis	Serology+ Molecular+	Symptomatic	Resolution
Yasuoka^3^	40 s	M	HTN, HLD	Diarrhea	—	Ileal and colonic thickening	Ileal and colonic ulceration	Intranuclear inclusion bodies	IHC+	Ganciclovir	Resolution
Ochiai^4^	74	F	None	Diarrhea, abdominal pain, edema	—	Ileal edema and thickening	Ileitis with ulceration	Ulcerative mucosa with intranuclear inclusion bodies	IHC+	Ganciclovir	Resolution
Perrineau^5^	35	M	None	Abdominal pain, vomiting, peritonitis	Leukocytosis, elevated CRP	Gastric and small bowel thickening	Mild gastritis and duodenitis	Chronic duodenitis with intranuclear inclusion bodies	Serology+ Molecular+ IHC+	Symptomatic	Resolution
Lalazar^6^	25	M	None	Abdominal pain, vomiting, fever	Leukocytosis, elevated LFTs	Hepatomegaly, splenomegaly	Esophagitis, thickened gastric folds with erosions	Foveolar hyperplasia with intranuclear inclusion bodies	Serology+ IHC+	Symptomatic	Resolution
Chen^7^	22	M	*H. pylori*	Epigastric pain, vomiting	Leukocytosis, elevated LFTs	Unremarkable	Erosive gastritis	Acute gastritis with intranuclear inclusion bodies	Serology+ Molecular+ IHC+	Ganciclovir	Resolution
Engjom^8^	30 s	M	None	Sore throat, edema, epigastric pain, fever	Elevated fecal calprotectin	Mildly enlarged spleen, enlarged mesenteric lymph nodes	Thickening of the gastric folds	Foveolar hyperplasia with intranuclear inclusion bodies	Serology+ PCR+	Ganciclovir followed by oral valacyclovir	Resolution
Suter^9^	34	M	None	Diarrhea, abdominal pain, fever	Elevated LFTs	Hepatomegaly	Thickening of the gastric folds with erosions	Chronic active gastritis	Serology+ IHC+ Culture+	Symptomatic	Resolution
Kraus^10^	76	M	Ulcerative colitis	Abdominal pain	—	—	Pancolitis with rectal sparing	Acute pancolitis	Serology+ IHC+ Culture+	Ganciclovir	Resolution
Nakase^11^	22	M	None	Edema	Leukocytosis	—	-	Edematous ilium	Serology+ IHC+	Symptomatic	Resolution
Bencharif^12^	23	M	None	Epigastric pain, diarrhea, edema, fever	Elevated LFTs	Lymphadenopathy, splenomegaly	Severe gastritis and thickening of gastric folds	Nonspecific gastritis and edematous gastritis	Serology+ Culture+	Symptomatic	Resolution
Nakazato^13^	38	M	None	Sore throat, fever	Elevated LFTs	Lymphadenopathy, hepatomegaly	Thickening of the gastric folds	Foveolar hyperplasia	Serology+	Symptomatic	Resolution
Underwood^14^	68	F	None	Stomatitis, diarrhea	Elevated ESR	—	Necropsy: Jejunal ulceration	Jejunitis with intranuclear inclusion bodies	—	—	Deceased from chest infection

CMV, cytomegalovirus; CRP, C-reactive protein; ESR, erythrocyte sedimentation rate; *H. pylori, Helicobacter pylori*; HLD, hyperlipidemia; HTN, hypertension; IHC, immunohistochemistry; LFTs, liver function tests; PLE, protein-losing enteropathy.

## CASE REPORT

**Figure 1.**
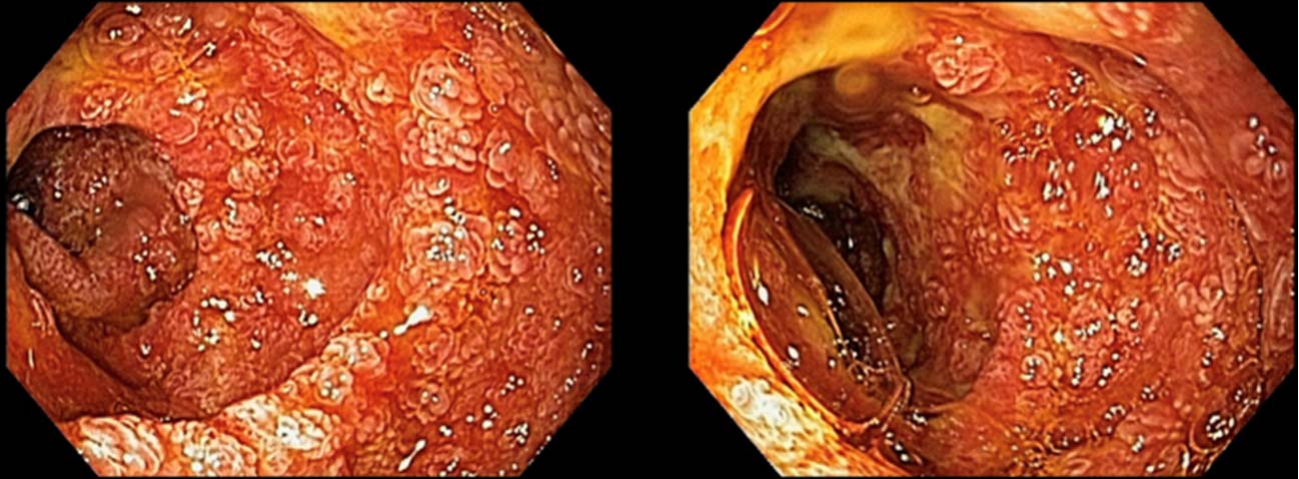
Erythema, erosions, friability, white plaques, ulceration, and scalloped folds in the whole examined duodenum compatible with ulcerative duodenitis.

**Figure 2.**
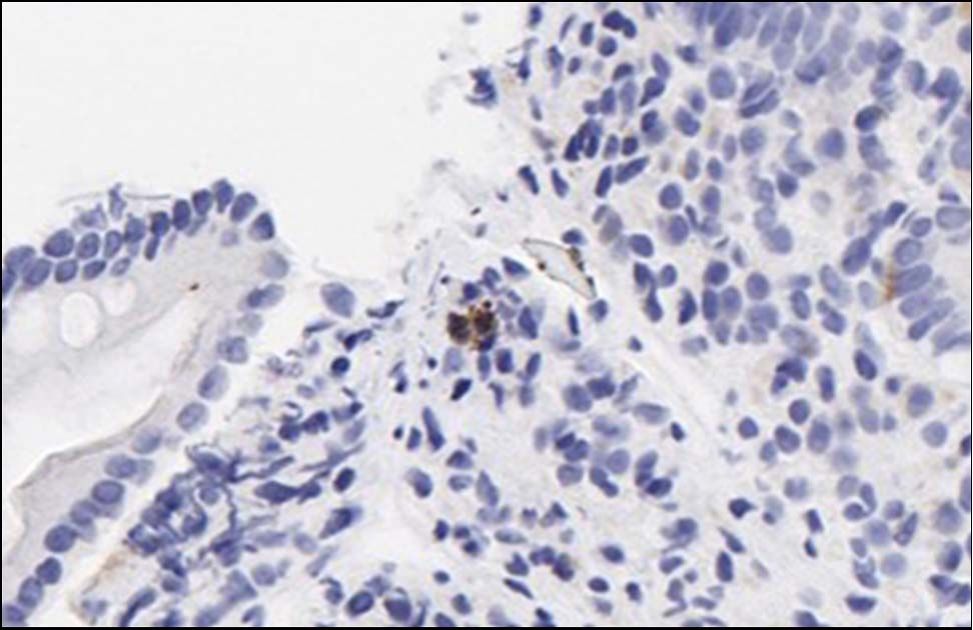
Immunohistochemical staining demonstrating cytomegalovirus-positive cells within the duodenal lamina propria (original magnification ×400).

**Figure 3.**
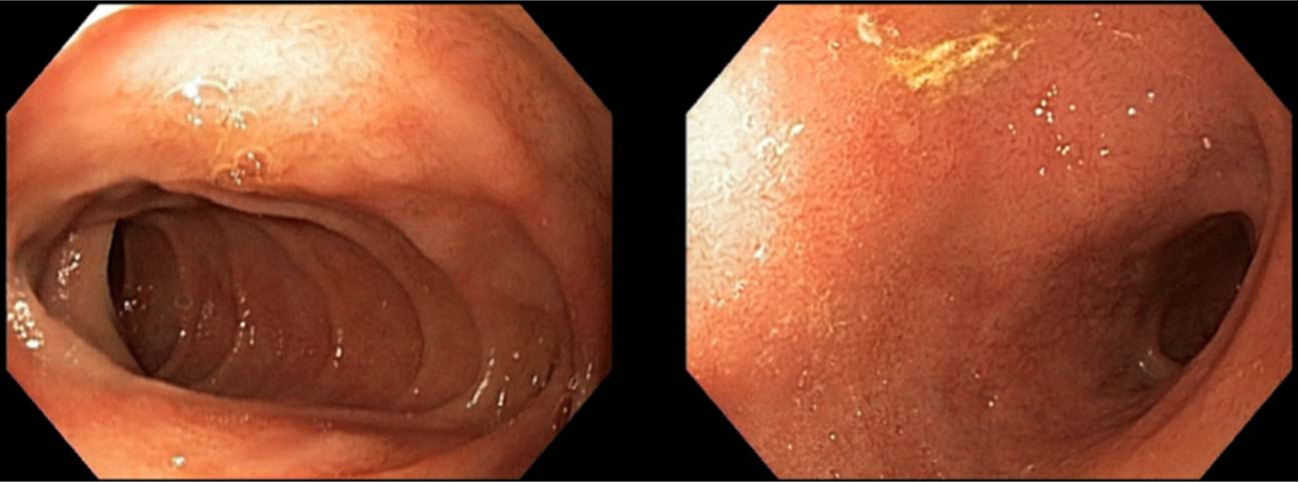
Mild erythema of the duodenum after valganciclovir treatment.

A previously healthy 60-year-old woman presented to the emergency department with intractable nausea, vomiting, and watery diarrhea for 2 weeks. She reported generalized fatigue during this period but denied fever, chills, myalgias, headache, dyspnea, vision changes, or sore throat. Physical examination was significant for diffuse abdominal tenderness, though the abdomen was soft and nondistended. He had no lymphadenopathy, enlarged tonsils, abnormal lung sounds, rash, or hepatosplenomegaly. Laboratory evaluation revealed mild leukocytosis (11.63 × 10^3^/μL) with normal C-reactive protein levels and liver function tests. Contrast-enhanced computed tomography showed mild splenomegaly with normal-appearing bowel and liver. Initial infectious workup, including *Clostridioides difficile* toxin, rotavirus and norovirus antigens, and bacterial stool cultures, was negative. Intravenous fluids were initiated; however, within a day, she developed anasarca, bilateral pleural effusions, and pronounced electrolyte disturbances despite aggressive replacement. Proteins including IgG, IgM, and albumin were low (IgG 228 mg/dL [reference range 650–1,600 mg/dL], IgM 41 mg/dL [reference range 50–300 mg/dL], albumin 1.7 g/dL [reference range 3.2–4.8 g/dL]), yet she displayed no signs of malnutrition, proteinuria, or synthetic liver dysfunction to explain the low protein levels. PLE was diagnosed with an elevated fecal alpha-1 antitrypsin (A1AT) clearance of 63.3 mL/day (positive >56 mL/day). Esophagogastroduodenoscopy at symptom onset showed esophagitis, erosive gastritis, and ulcerative duodenitis (Figure 1). Histopathology revealed chronic esophageal inflammation, erosive gastric antral mucosa without *Helicobacter pylori*, and ulcerative duodenitis with increased intraepithelial lymphocytes. Comprehensive evaluation for the underlying etiology of PLE returned with positive CMV testing (CMV IgM 62.9 AU/mL [positive >34.9 AU/mL], CMV IgG 2.4 U/mL [positive >0.69 U/mL], CMV quantitative DNA polymerase chain reaction 46,100 IU/mL [positive >200 IU/mL]). She was diagnosed with PLE due to CMV, which was later confirmed by CMV-specific immunohistochemistry (Figure 2). CMV immunohistochemical staining was positive only in duodenal tissue, though colonoscopy was not performed. She completed 3 weeks of valganciclovir with resolution of her symptoms. Follow-up laboratory studies demonstrated resolution of leukocytosis (5.3 × 10^3^/μL), normalization of albumin (3.6 g/dL) and IgM levels (57 mg/dL), and undetectable CMV DNA by polymerase chain reaction. IgG levels remained mildly decreased but showed significant improvement (523 mg/dL). Repeat endoscopy showed only mild duodenitis with no erosions or ulcerations (Figure 3).

## DISCUSSION

In healthy individuals, the daily loss of protein through the gastrointestinal tract plays a minor role in overall protein metabolism; it contributes to only 1%–2% of the total serum protein and less than 10% of the total albumin pool.^15^ However, in cases of PLE, gastrointestinal protein loss can reach up to 60% of the total albumin levels. Serum proteins most impacted by this process are those with slower turnover rates, including albumin, immunoglobulins, and ceruloplasmin. Longer half-lives prevent compensation for such losses.^16^

Diagnosis of PLE begins with exclusion of more common causes of hypoproteinemia, including malnutrition, hepatic disease, and renal disease. Once these potential causes are ruled out, an A1AT clearance can be measured. A1AT is a protein synthesized in the liver that is not actively secreted or absorbed, it has a molecular weight similar to albumin, and it remains intact as it passes through the gastrointestinal tract. These properties allow for detection in feces. Normal A1AT clearance is less than or equal to 21 mL/day or less than or equal to 56 mL/day with diarrhea. An increase in A1AT clearance is indicative of PLE.^1^

CMV-associated PLE in immunocompetent adults is seldom reported. The gold standard for diagnosis of CMV-related gastrointestinal disease relies on immunohistopathology. Gastrointestinal disease may be focal and patchy, so multiple biopsies may be needed to confirm the diagnosis. In addition, visualization of CMV inclusion bodies necessitates that tissue biopsies be taken from the ulcer edge or base and, therefore, is easily missed. Serum CMV testing may assist with early detection and monitoring of response to therapy if positive, but immunohistopathology remains the gold standard for diagnosis.^17^

The treatment of PLE typically includes dietary supplementation and treatment of the underlying disease. While treatment with antivirals is recommended for CMV enteritis in immunocompromised individuals, its role in immunocompetent individuals is not well defined. A majority of immunocompetent patients with CMV disease recover without intervention. Symptomatic and supportive nutritional therapy such as antinausea medications, electrolyte replacement, and a high-protein diet often result in improvement of symptoms.^6^ In similar cases of CMV-associated PLE, patients' symptoms resolved after 2 to 4 months of supportive treatment.^2,5,6,9,11–13^ Practically, the severity of CMV disease must be balanced against the risk of medication toxicity. In severe disease that is nonresponsive to supportive treatment, patients may benefit from antiviral therapy. In our case, the patient's CMV disease required hospitalization with persistent electrolyte derangements, thus antiviral treatment was initiated with significant clinical improvement.

In conclusion, CMV-associated PLE may occur in immunocompetent adults and manifest as anasarca with severe and prolonged gastrointestinal symptoms. Prompt recognition of anasarca with concurrent low serum protein levels as manifestations of a PLE will hasten diagnosis of CMV-associated enteritis and enhance patient outcomes.

## DISCLOSURES

Author contributions: N. Mayer participated in patient care, reviewed the literature, and contributed to the writing of the manuscript. N. Bhargava, D. Patel, M. Kaplan, and V. Kata participated in patient care and reviewed and edited of the manuscript. D. Errampalli and T. O'Connor contributed to patient care, endoscopic evaluation of the patient, and reviewed and edited of the manuscript. T. Betlej evaluated the pathology slides and reviewed and edited of the manuscript. N. Bhargava is the article guarantor.

Financial disclosure: The authors have no conflicts of interest to declare.

Previous presentation: Presented as a poster at the ACG 2024 Annual Scientific Meeting, October 2024, Philadelphia, PA.

Informed consent was obtained for this case report.

## ABBREVIATIONS:

A1AT: alpha-1 antitrypsin
CMV: cytomegalovirus
IgG: immunoglobulin G
IgM: immunoglobulin M
PLE: protein-losing enteropathy
